# Possible changes to arable crop yields by 2050

**DOI:** 10.1098/rstb.2010.0153

**Published:** 2010-09-27

**Authors:** Keith W. Jaggard, Aiming Qi, Eric S. Ober

**Affiliations:** Rothamsted Research, Broom's Barn Research Centre, Higham, Bury St Edmunds, Suffolk, UK

**Keywords:** carbon dioxide, ozone, climate change, plant breeding, yield gap, pest and disease control

## Abstract

By 2050, the world population is likely to be 9.1 billion, the CO_2_ concentration 550 ppm, the ozone concentration 60 ppb and the climate warmer by *ca* 2°C. In these conditions, what contribution can increased crop yield make to feeding the world?

CO_2_ enrichment is likely to increase yields of most crops by approximately 13 per cent but leave yields of C4 crops unchanged. It will tend to reduce water consumption by all crops, but this effect will be approximately cancelled out by the effect of the increased temperature on evaporation rates. In many places increased temperature will provide opportunities to manipulate agronomy to improve crop performance. Ozone concentration increases will decrease yields by 5 per cent or more.

Plant breeders will probably be able to increase yields considerably in the CO_2_-enriched environment of the future, and most weeds and airborne pests and diseases should remain controllable, so long as policy changes do not remove too many types of crop-protection chemicals. However, soil-borne pathogens are likely to be an increasing problem when warmer weather will increase their multiplication rates; control is likely to need a transgenic approach to breeding for resistance. There is a large gap between achievable yields and those delivered by farmers, even in the most efficient agricultural systems. A gap is inevitable, but there are large differences between farmers, even between those who have used the same resources. If this gap is closed and accompanied by improvements in potential yields then there is a good prospect that crop production will increase by approximately 50 per cent or more by 2050 without extra land. However, the demands for land to produce bio-energy have not been factored into these calculations.

## 1. Introduction

By 2050 it is predicted that there will be between 8.0 and 10.4 billion people on earth, with a median value of 9.1 billion (http://esa.un.org/unpp). If all of these people are to be fed sufficiently, total food consumption will have to increase by 50–70% (Smil 2005; FAO 2009). How much of a contribution can increased yield per unit area make? Many studies have addressed this problem, and the large ones have done so from the viewpoint of agro-ecology and climate science or of socio-economics (Fisher *et al*. 2005; Nelson *et al*. 2009). This review examines the question from the viewpoint of crop physiology and agronomy.

By 2050, assuming the A1B world development pathway from the Special Report on Emissions Scenarios (Nakićenović & Swart 2000), it is predicted that the carbon dioxide concentration [CO_2_] will have risen from today's value of approximately 370 to 550 ppm. This, in combination with other changes in the atmosphere, is likely to change the earth's climate, making it warmer by an average of 1.8°C (Meehl *et al*. 2007). This warming will increase the evaporation of water from wet surfaces and from plants, leading to increased but more variable precipitation. At present, the amount and seasonality of precipitation in any region can only be predicted with a great deal of uncertainty. The concentration of ozone [O_3_] will also increase as a result of industrialization and this will have a negative impact on crop growth and productivity: this has been assessed by reviewing recent literature.

We have selected 11 arable crops, and assess the extent to which changes in yields might contribute towards an increase in the amount of food available. These crops (table 1) represent the principal types of photosynthesis used by plants, C3 (wheat, rice, soya, sunflower, oilseed rape, potato, sugar beet and dry bean) and C4 (maize, sugar cane and sorghum). Two of the crops (soya and dry bean) are legumes and fix nitrogen, as does much sugar cane in Brazil (Döbereiner 1997). Wheat, rice, maize and sorghum occupy 83 per cent of the world's total cereal area. Together, these crops occupy 56 per cent of the world arable area. We have attempted to assess the extent to which crop yield changes might contribute to feeding the world's population using the literature to assess the probable yield changes and by making an analysis akin to that of Ewert *et al*. (2005). These authors estimated future changes in productivity of a range of crops in 17 European countries from trended values of current yields modified by relative changes owing to climate change, increasing [CO_2_] and technology development. Future technology development effects were estimated from historic trends in relative changes of national yields and progressed into the future.

**Table 1.** World production statistics for major crop types (2007). Data accessed in November 2009 from Food and Agriculture Organization: http://www.fao.org.

	million ha	million tonne	tonne ha^−1^
wheat	214.2	606	2.8
maize	158.0	792	5.0
rice	155.8	660	4.2
sorghum	46.9	63	1.4
soya bean	90.2	221	2.4
dry beans	26.5	18	0.7
rape	30.8	51	1.6
sunflower	21.5	27	1.2
cane sugar	22.7	107	4.7
beet sugar	5.2	44	8.4
potatoes	18.5	309	16.7

## 2. Carbon dioxide concentration

The [CO_2_] in the atmosphere can have a large impact on the rate of photosynthesis, particularly of C3 plants. This effect is used commercially in tomato production where the air in glasshouses is enriched to greatly increase yield. However, [CO_2_] also affects water use by plants because high concentrations cause partial closure of the stomata. The magnitude of its effects on dry matter production depends upon the illumination conditions, water availability, N supply and the transport and storage of the photosynthates. This complexity means that the interpretation of controlled environment studies (where enriching the air with CO_2_ is relatively straightforward) is fraught with difficulty. To overcome this, free air carbon dioxide enrichment (FACE) experiments have been made in the last two decades. In these, crops are grown to maturity in the field in either an ambient atmosphere or one enriched with CO_2_. Most of these studies used an atmosphere close to 550 ppm CO_2_, and these are considered here. Studies with grain crops were reviewed by Long *et al*. (2005*a*), who found that the average yield increase of C3 species was 11 per cent. In FACE experiments in Germany Manderscheid & Weigel (2006) grew two cycles of a three-year rotation of winter barley, sugar beet and winter wheat using adequate applications of nitrogen fertilizer and measured yield increases of 13, 15 and 7 per cent, respectively, in response to [CO_2_]. In Italy, FACE experiments with potato (Miglietta *et al*. 1998; Magliulo *et al*. 2003) produced much larger yield increases (29%, 32% and 54%) in response to increased [CO_2_]. Is it significant that the two C3 species (potato and tomato) with large responses to increased [CO_2_] are both members of the *Solanaceae?*

Long *et al*. (2005*a*) also reported FACE results for maize and sorghum (C4 species) where there were no significant responses to enrichment. All these yield results, except those for potato, are less than anticipated from earlier reviews, most of which were based on studies in controlled conditions (Amthor 2001; Kimball *et al*. 2002). The responses to enriched atmospheres in the FACE experiments are also smaller than those that have been used in most crop-growth models. Possible reasons for the smaller increases are that field-grown crops have canopy architectures that are not optimized for the efficient use of radiant energy, and that feedback repression of photosynthesis occurs because the plants are incapable of transporting or storing sugars at the greater production rate of the enriched atmospheres i.e. they are sink limited.

The [CO_2_] affects the water economy of crop plants. Increased [CO_2_] increases the rate at which this gas diffuses into leaves through the stomata, relative to the rate at which water vapour diffuses out. Because the extra CO_2_ increases the rate of dry matter production of C3 plants, this change in relative diffusion rates also increases the water use efficiency (WUE), the amount of dry matter produced per unit of water transpired. An increase in the [CO_2_] also causes a decrease in the aperture of the stomata, which reduces the rate of water consumption. In the FACE experiments with potatoes this effect was large: CO_2_ enrichment increased tuber yield by 43 per cent, decreased water consumption by 11 per cent and consequently increased WUE by approximately 70 per cent (Magliulo *et al*. 2003). In sugar beet, the amount of water consumed during the growing season was reduced by 20 per cent while yield increased by 8 per cent (Manderscheid *et al*. 2010). The impact of this water economy on yield is difficult to determine because, to date, it has not been possible to conduct FACE experiments with both warmed air and CO_2_ enrichment. However, it is clear that this effect of CO_2_ on water consumption can only have a positive impact on yield because in many situations crop yields are water-limited, and this effect has not been built into the simulations of future food production made so far.

In most FACE experiments the plants were supplied with adequate water and nitrogen fertilizer. However, experiments with wheat (Kimball *et al*. 1999), rice (Kim *et al*. 2003) and a cereal and beet rotation (Burkart *et al*. 2009) compared enrichment responses at inadequate and sufficient levels of N supply. In all cases the relative response to enriched [CO_2_] was either enhanced or unchanged when the N amount was inadequate. Similar responses were measured in clover; plants with plentiful nodules produced smaller responses than plants with few nodules (Hartwig *et al*. 2002). In future, if for financial or environmental reasons, N fertilizer use is further restricted, the enriched CO_2_ atmosphere should help to limit the negative impact on crop yield.

## 3. Ozone concentration

Ozone concentrations [O_3_] in the industrialized countries of the Northern Hemisphere have been rising at between 1 and 2 per cent per year (Chameides *et al*. 1994). The surface [O_3_] has now reached a global mean of approximately 50 ppb (8 h summer seasonal average; Fiscus *et al*. 2005). Nearly a quarter of the Earth's surface is at risk of experiencing concentrations in excess of 60 ppb during mid-summer. Yield reductions owing to ozone pollution can begin at concentrations as small as 20 ppb (Ashmore 2002). The IPCC Fourth Assessment Report projects an increase in surface [O_3_] across the globe of 20–25% by 2050 (Meehl *et al*. 2007). Long *et al*. (2005*b*) estimated that a 20 per cent increase will decrease yields relative to today by 5, 4, 9 and 12 per cent for maize, rice, wheat and soya, respectively. Potatoes have suffered a yield reduction of 5 per cent (Craigon *et al*. 2002). A meta-analysis by Feng & Kobayashi (2009) found that probable yield reductions by 2050 were 8.9, 9 and 17.5 per cent for barley, wheat and rice, but were 19.0 and 7.7 per cent for bean and soya bean. These projections were made on the basis of studies in open-topped chambers in the field. Only two FACE studies (Morgan *et al*. 2003; Shi *et al*. 2009) have been reported with ozone enrichment: the first reduced soya yield by 20 per cent, the second produced rice yields that were unaffected (two inbred cultivars) or were reduced by 15 and 17.5 per cent (two hybrid cultivars).

Changes in yield as a consequence of rising [O_3_] have not been built into recent projections of global food production under climate change (Gitay *et al*. 2001; Parry *et al*. 2004; Nelson *et al*. 2009). The predicted yield changes are rather variable even within species, but they are all reductions. By 2050 the impact of rising [O_3_] is likely to eliminate most of the yield increase owing to increasing [CO_2_] in C3 crops, and cause a yield decrease of at least 5 per cent in C4 species. However, the studies with rice indicate that there is scope to breed for reduced O_3_ sensitivity.

## 4. A changed climate

A consequence of the increase in the [CO_2_] and the concentration of other gases in the atmosphere is that the world is expected to get warmer, by about 1.8°C as an annual average by 2050, and by rather more over land (Gornall *et al*. 2010). This will be accompanied by changes in precipitation, more than today in some places, and less in others.

We did not have access to observed daily weather data at sufficient international locations to use crop-growth models to simulate the impacts of future climates on yields. Instead, we have relied on published results. There have been three large studies. The first was summarized by Parry *et al*. (2004): it used climate simulations from general circulation models (GCMs) developed in the 1980s. The CERES and SOYGRO models were used to simulate the growth of wheat, rice, maize and soya bean at 118 locations around the world, with and without a CO_2_ effect on growth. In broad terms for 2050 and in the absence of the CO_2_ effect on growth, the findings were:

— In low latitudes crop yields are likely to decrease, mainly owing to increased temperature which shortens the period for grain filling and sometimes stresses the plants at the time of flowering and seed-set.
— At higher latitudes yields are likely to increase slightly as warmer weather allows longer growing seasons.

The second (Fisher *et al*. 2005) used five GCMs, the world soil map, agro-ecological zoning, simulations of today's climate and scenarios from five GCMs to predict production of cereal crops on 5′ × 5′ grid across the globe, driven by predicted changes in socio-economics and world food trade. They predicted that world cereal production will increase from 1.8 Gt today to between 3.7 and 4.8 Gt by 2080. Much of this increase will be the result of cropping on an additional 320 million ha in the Northern Hemisphere.

The third and most recent study, by the International Food Policy Research Institute (Nelson *et al*. 2009), modelled maize, wheat, rice, groundnuts and soya beans at 0.5° intervals using simulations of the current weather around the world, based on monthly average values for the period 1950–2000 and decision support system for agrotechnology transfer (DSSAT) crop models. These results were applied to other crops: C4 species were assumed to behave like maize, C3 types were assumed to behave like wheat, rice and soya. The climate simulations were generated by models from the National Center for Atmospheric Research (NCAR) and from Commonwealth Scientific and Industrial Research Organization (CSIRO), using the A2 global development path. The [CO_2_] in this pathway is similar to A1B by 2050. The NCAR simulations for 2050 predict an extra 10 per cent precipitation on land whereas the CSIRO simulations predict an extra 2 per cent: the HadCM3 predictions are for an increase of about 4 per cent over cropped land (Gornall *et al*. 2010). The NCAR simulations also indicate larger temperature increases than the CSIRO or HadCM3 models, especially in the Northern Hemisphere. The results of the estimated yield changes, averaged for the two climate simulators and without CO_2_ fertilization, are presented in table 2 for wheat, rice and maize. In most cases the yield reductions owing to climate change were more serious when the NCAR simulations were used, or the yield increases were smaller; the average difference was 3 per cent. This happened whether the crops were irrigated or rain-fed: this is surprising since the climate simulations with the most precipitation would be expected to produce the larger rain-fed yield. In almost all cases the yield reductions were more serious in developing countries. Of necessity, almost all of the input data for the yield models were simulated and this raises questions about the reliability of the output. Unfortunately, Nelson *et al*. (2009) give no indication of whether their yield simulations for today's climate are similar to reality or not. Certainly the assumptions made to determine crop sowing date are such that rain-fed crops would seldom be sown in eastern England, Canada, Russia or the western half of the USA.

**Table 2.** Mean percentage yield changes by 2050, in the absence of a CO_2_ fertilization effect, estimated using climate changes simulated from two simulators and yields simulated with the DSSAT crop-growth models. Source: data from simulations by Nelson *et al*. (2009).

crop	developing countries	developed countries
maize irrigated	−2.4	−5.0
maize rain-fed	−0.3	−2.6
rice irrigated	−16.5	−4.5
rice rain-fed	−0.9	14.4
wheat irrigated	−31.3	−5.3
wheat rain-fed	−1.3	2.8

The variations between predicted outcomes of climate change arising from different climate simulators and different modelling methods are illustrated by comparing the results in table 2 for rice with the results produced by Masutomi *et al*. (2009). These authors used a model, agro-ecological zones for Asia and 18 GCMs to conclude that by 2050 and without a CO_2_ fertilizing effect, rice yields would decrease by an average of 8 per cent, not the 16 per cent implied in developing countries in table 2. These authors ascribed most of this yield reduction to warmer winters that would affect the impact of weeds, pests and diseases. However, no consideration was given to the likelihood that these impacts would be controlled by farmers.

The large differences between the predicted outcomes for these climate simulators and modelling approaches illustrates just how tentative we should be about the predicted outcome of climate change, albeit that most studies agree that yield will be reduced.

Extreme weather events are more likely to happen in the changed climate of the future (Gornall *et al*. 2010). It is obvious that the severity and frequency of drought will affect crop production: the extreme heat effects are less obvious recently. Gornall *et al*. (2010) show that over much of the world's crop land, today's 1-in-20 year event is likely to be approximately 3°C hotter by 2050. Increases of this sort will have serious negative impacts if they occur during the flowering stages of many crops (Wheeler *et al*. 2000); whether the temperature sensitivity of these stages is highly conserved within a species is not clear, so breeding for tolerance may be difficult. Furthermore, plant breeders are unlikely to select for tolerance to an event that is predicted to be rare. Farmers may have to adapt by growing more tolerant species. Unfortunately, the species that seem best adapted to high temperatures have received little attention from international plant breeders until now.

**Figure 1.**
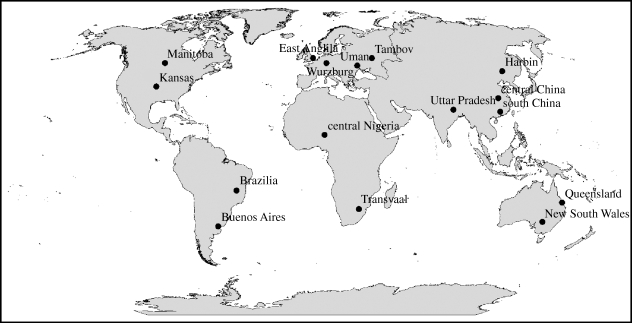
Selected sites for weather data and crop yield assessment in different regions.

Yield simulations made on the basis of predicted future climate seldom simulate in a realistic way the possible impacts of pests, diseases or weeds (whose impacts might become more or less serious) or take account of many of the possible adaptations that plant breeders and farmers might make in response to climate change. In an attempt to make a qualitative assessment of some adaptations that might occur, we examined the simulated weather data from 16 regions that represent major zones of arable crop production around the world (figure 1). One of the regions is East Anglia, chosen not because it represents a large production area, but because its characteristics are familiar to us. The percentage of world production of major crop types within these countries is presented in table 3. The UK Meteorological Office provided daily weather simulation output from HadCM3 for these locations for 10-year time slices centred on 2000 and 2050. These simulations are for grids where little of the area is sea.

**Table 3.** Percentage of world production for selected crops in selected countries/regions averaged over 2003–2007. Values less than 1% have been omitted. Data accessed in November 2009 from Food and Agriculture Organization (FAO): http://www.fao.org.

crop	EU	Argentina	Australia	Brazil	Canada	China	India	Nigeria	Russia	S. Africa	Ukraine	USA	% of world
wheat	21.2	2.5	3.2		3.9	16.3	11.6		7.3		2.2	9.4	77.7
maize	8.3	2.4		6.2	1.3	19.2	2.2			1.3	1	40	81.9
rice				1.9		28.6	21.7					1.5	53.8
sorghum		4.3	2.9	2.9		4.3	12	15				17.2	58.6
soya bean		18.3		25.1	1.4	7.5	4.1					37.3	93.7
dry bean		1.2		16.8	1.6	8.9	16.8					5.6	51
rape seed	32.8		2.8		18.3	26.3	14.2					1.4	95.8
sunflower	21.7	12.5				6.2	4.4		20	2	15	4.4	86.2
sugar cane		1.5	2.6	32.1		6.8	19.7			1.4		1.9	66.2
sugar beet	50.1					3.2			9.9		6.9	11.6	81.7
potato	20.2			1.0	1.6	20.4	7.4		11.8		6.2	6.4	75

**Figure 2.**
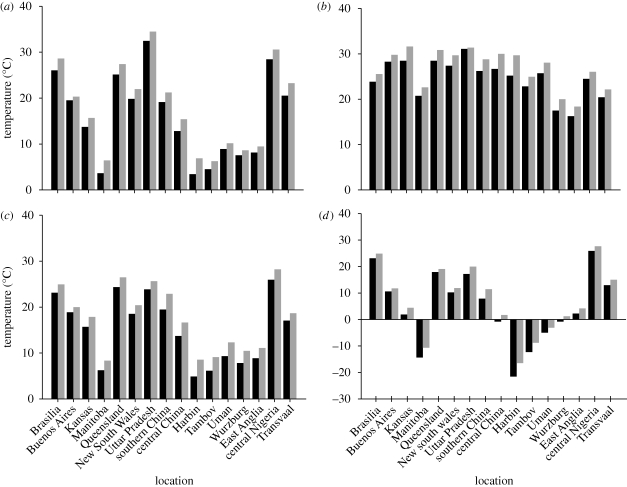
Seasonal mean temperatures at selected sites (see figure 1) in decades centred on 2000 (filled black bar) and 2050 (filled grey bar). The Northern Hemisphere (*a*) spring is March, April and May; (*b*) summer is June, July and August; (*c*) autumn is September, October and November and (*d*) winter is December, January and February. The allocation of the months is reversed in the Southern Hemisphere. The data are the means of 10 years' daily simulations generated by HadCM3.

**Figure 3.**
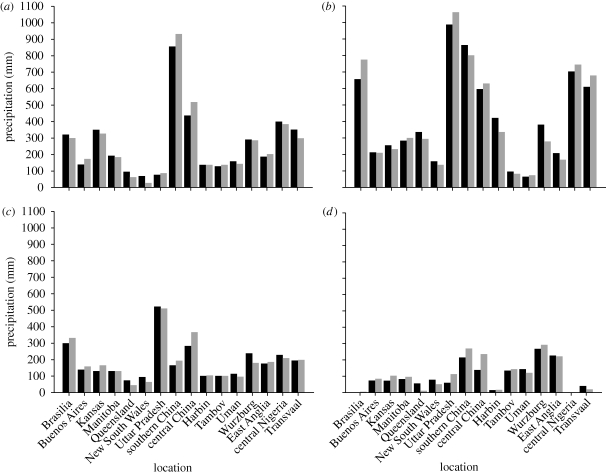
Seasonal total precipitation at selected sites (see figure 1) in decades centred on 2000 (filled black bar) and 2050 (filled grey bar). The Northern Hemisphere (*a*) spring is March, April and May; (*b*) summer is June, July and August; (*c*) autumn is September, October and November and (*d*) winter is December, January and February. The allocation of the months is reversed in the Southern Hemisphere. The data are the means of 10 years' daily simulations generated by HadCM3.

The temperature and precipitation at the 16 locations are summarized in figures 2 and 3. Clearly, all locations are anticipated to become warmer. For example, mean spring temperatures in Manitoba are predicted to increase from 3.7°C to 6.4°C: similar increases are predicted for Harbin, northern China and Tambov in Russia. Similarly, during autumn in Harbin, mean air temperatures are predicted to rise from 4.8°C to 8.8°C. These shifts are large enough for the growing seasons of crops like soya, maize, potato and beet to be lengthened considerably, and in turn this should generate large yield increases, provided there is sufficient water for the crops to avoid serious drought. Unfortunately, the summer in Harbin is predicted to become drier, with rainfall decreasing from 422 mm to 338 mm (figure 3). Similarly, in New South Wales, Australia, average spring rainfall is predicted to decrease from 70 mm to 29 mm and in Germany on the loess soils, summer rain will decrease from 380 mm to 280 mm. In the first case, this will significantly affect the chance that crops can be established successfully, and in the second it will increase the risk of drought for crops that grow throughout the summer, like maize and sugar beet.

## 5. Improving technology

The yields of arable crops in the developed and developing countries of the world changed enormously in the last half of the twentieth century. Average yields of wheat in the UK rose from 3 to 8 t ha^−1^ while the world average has risen from 1.08 to 2.7 t ha^−1^. Reilly & Fuglie (1998) showed that the average yields of 11 crops in the USA had increased by between 1 and 3 per cent per year during the last half century and that the trend was linear or exponential, showing no sign that the rate was slowing down. A large study by Hafner (2003) showed that national average yields of wheat, rice and maize in 188 countries were mostly increasing, that the increases had been predominantly linear, and that the biggest producers' yields had increased at more than 33.1 kg ha^−1^ yr^−1^. This rate of yield improvement is required if *per capita* consumption is to remain at current levels by 2050. In the developed and developing countries much of this increase has been due to the use of nitrogen fertilizer, crop-protection chemicals and responsive varieties. The yield increases delivered by optimizing the use of N fertilizer and by controlling pests, disease and weeds cannot be repeated: if a disease is controlled and yield increases, then this cannot be repeated to achieve another yield increase. This begs the question ‘Are yield increases in the past any guide to increases in the future?’.

### (a) Plant breeding

Silvey (1994) studied UK national cereal yields over recent decades and concluded that the proportion of the yield change attributed to plant breeding was 47 per cent for wheat and 55 per cent for barley. This compares with 58 per cent for maize in Minnesota (Reilly & Fuglie 1998) and 50 per cent for USA as a whole (Duvick & Cassman 1999). Nevertheless, and despite a new world record wheat yield of 15.6 t ha^−1^ set in New Zealand in 2010 (http://www.fwi.co.uk/Articles/2010/03/17/120390/Farmer-topples-his-own-wheat-world-record.htm) questions are being asked about whether these historic improvements have continued in the last 10–15 years. Spink *et al*. (2009) presented data from UK wheat and oilseed rape variety trials from 1997 to 2006 which show almost no upward trend. Similar evidence can be produced for potato (Allen *et al*. 2005). However, these plateaux in the trends are still of short duration and could result from annual climate variations. In sugar beet in the UK, Jaggard *et al*. (2007) showed that sugar yield increases since 1976 were mostly the result of warmer springs and that only about 30 per cent of the improvement was the result of technological advance, including plant breeding.

Evidence like this has fuelled the debate about the extent to which plant breeders are reaching a yield ‘ceiling’. To increase yield, plant breeders must increase the combination of solar energy capture by photosynthetically active parts of plants, improve radiation use efficiency (RUE) or shift dry matter distribution in favour of the harvestable part of the plant (harvest index, or HI). For example, much of the advance made with wheat has been achieved by shifting HI (Austin 1999), and little progress has been made to increase rates of photosynthesis (Richards 2000) or ability to tolerate drought. HI is thought to be at about optimum now and this has led to the perception that perhaps the ceiling yield has nearly been achieved. Spink *et al*. (2009) discussed this and referred to blueprints for wheat and oilseed rape that could produce 19 t ha^−1^ and 9 t ha^−1^, respectively, in ideal agronomic conditions in the UK. Compared with today's crops, much of the increase would be owing to increased light capture achieved by breeding for delayed senescence. This will be especially important in future, to counteract the effect of a warmer climate that would make grain crops mature earlier.

Enrichment of the atmosphere with CO_2_ might offer the plant breeder the opportunity to raise the yield ceiling by increasing RUE and WUE, which are difficult breeding targets. The gains in yield made by almost all C3 crop species in the FACE experiments were smaller than anticipated from studies in controlled conditions. It has been speculated that this is mostly caused by the inability of today's crops to either transport or store the sugars at a rate that keeps pace with the production capacity of leaves that are operating in the enriched atmosphere: the capacity of the sink limited the yield. There is debate about whether grain crops are limited by the sink capacity in today's conditions (Sinclair & Jamieson 2008) and indications that beet crops growing in a CO_2_-enriched atmosphere will be limited as well (Manderscheid *et al*. 2010). As the [CO_2_] increases, plant breeders will gradually, and perhaps inadvertently, select for lines that have less of a sink limitation (Sun *et al*. 2009).

In C3 crops, leaf photosynthesis is saturated at radiant flux densities of between a quarter and half of full sunlight, therefore any solar energy intercepted at above this level is wasted. Another approach that has been postulated to increase RUE is to manipulate canopy architecture so that, while the sun is bright, more of the canopy is illuminated at moderate intensity and less is light-saturated (Long *et al*. 2005*a*). This can be done by making the uppermost leaves nearly vertical, so that they are not light-saturated, while the lower leaves are almost horizontal to ensure that almost all the light is intercepted. This approach has been a major factor in improving the productivity of rice (Beadle & Long 1985), but it has fallen from fashion. Nevertheless, it has the potential to increase RUE by as much as 40 per cent at mid-day in full sunlight (Long *et al*. 2005*a*). However, like the CO_2_ effect, in order to benefit from this change, today's crops would need less sink limitation.

Targets like changing sink capacity and canopy architecture can be tackled, if necessary, by conventional plant breeding. More exotic approaches such as engineering C4 photosynthesis into C3 species are likely to be much more complex and difficult to deliver (Hibberd *et al*. 2008)—C4 species not only have different photosynthesis biochemistry, but they also have different leaf anatomy (Kranz anatomy) which is crucial to their efficient functioning. This anatomy is responsible for increasing the [CO_2_] around the mesophyll cells by several times its ambient concentration. A successful attempt to improve C3 crop yields by engineering them so that they use C4 photosynthesis would also have to engineer a version of the Kranz anatomy (Long *et al*. 2005*a*). A more successful strategy would be to extend the environmental range of existing C4 crops. Detailed descriptions of the opportunities and possible problems of breeding wheat with more productive biochemical pathways were reviewed by Reynolds *et al*. (2009).

In addition to concentrating on raising the potential yield, plant breeders will have to continue or even increase the attention they give to breeding crops for resistance to pathogens in order to increase the obtainable yield and its stability. This is especially important in the developed countries, many of which are restricting the types and amounts of pesticide permitted to be applied. Increased effort to breed for resistance to pests and pathogens is likely to divert resource applied to breeding for yield potential, reducing the pace at which yield can be improved. Furthermore, it is not uncommon that new sources of genetic resistance confer a yield penalty when crossed with elite material, and it takes some time to overcome this drag effect (Fisher & Edmeades 2010).

Another consideration is to assess the role of minor or under-used crop species. A large proportion of human caloric intake depends on a few graminaceous species (rice, wheat, maize), and this will not change significantly. However, these species represent a small fraction of the biological diversity of edible plants, and some species could become more important in the future. For example, cassava is a staple food for millions in tropical and subtropical regions, yet investment in improvements to this crop pales in comparison to the major ones. Likewise quinoa, a nutritious C4 grain of South American origin, could make a larger contribution with further development in genetics and agronomy. Globally, the minor, under-used species are likely to have only a small effect on feeding the billions, but locally, the impact of higher yields could be significant.

In conclusion, there is little reason to suppose that crops are approaching a yield ceiling, and every reason to expect that yields will increase as new varieties are introduced that are adapted to the changed, CO_2_-enriched environment. A large proportion of the yield increases that will be required to feed the world's population must be delivered via plant breeders. They will need to make advances as quickly as in the past, if not faster, and they will therefore need all the tools that biotechnology can provide: genomics and bioinformatics are likely to be of paramount importance (Phillips 2010). There is an implicit danger in too great a reliance on potential biotechnological breakthroughs to provide a ‘second green revolution’ (Sinclair *et al*. 2004). While it is possible that single transgene events could radically alter plant performance in a positive way under field conditions, diverting resources and focus from conventional breeding could slow the rate of yield increases.

### (b) Crop nutrition

Improved crop nutrition, particularly the provision of nitrogen fertilizer, has made huge increases in yields in developed economies. For example, in wheat in the UK, the optimum dose of nitrogen fertilizer, now about 200 kg N ha^−1^, increases yield about two-fold. Between 1950 and 1980, average N dressings for winter wheat increased from 50 to 180 kg ha^−1^ but have risen only slowly since then. Today it is rare for crops in countries with well developed arable agriculture to receive sub-optimal doses of N fertilizer, and applications are falling slightly as farmers fine-tune their agronomy. However, table 4 clearly shows that arable land in many regions is either being mined for nutrients (which is not sustainable) or is producing suboptimal yield. Fertilizer use in East Asia seems lavish, but more than one crop per year is common in parts of that region. The transition countries (the former Soviet Union) used far less fertilizer during the period of restructuring and reorganization, but its use is increasing again. Farmers in sub-Saharan Africa could increase their production considerably if they had access to fertilizer and the technology to use it appropriately: the limitation is probably poverty. In much of the world there is scope to increase fertilizer application per hectare by 50 per cent, and this would produce significant yield gains and slow or even prevent deterioration of land quality.

**Table 4.** Annual average nutrient applications (N + P_2_O_5_ + K_2_O) to arable land (1997–1999). Data from FAO (Bruinsma 2003).

region	nutrient application kg ha^−1^
East Asia	195
industrialized countries	118
South Asia	102
Near East & N. Africa	71
Latin America	56
transition countries	28
sub-Saharan Africa	5
world	92

In future, as yields rise, so will nutrient off-takes, and these nutrients will need to be replaced if the agriculture is to be sustainable. There is scope to increase the proportion of N fertilizer that is taken up by plants. Some crops leave a large proportion of the soil-applied fertilizer in the soil at the end of the growing season, where it represents a waste of resource to the farmer and a pollutant to water and/or air. Crops that are grown for their protein content, like bread-making wheat, will need more N in their grain in future if their yields are to continue to rise. More efficient ways to apply this N will be needed so that it does not get left in the soil, where it is prone to leaching and a cause of water pollution. Plant breeders and agronomists have started to search for ways to improve uptake and N use efficiencies.

### (c) Crop protection

Crop-protection chemicals (herbicides, insecticides and fungicides), like nutrients, have played a huge part in increasing and sustaining the yields of arable crops in industrialized countries. Oerke & Dehne (1997) analysed literature and field experiments from around the world and calculated the percentage of potential losses prevented by control measures, i.e. the efficacy of control. In 1991–1993 efficacy reached only 34–38% in rice, wheat and maize but was 43 per cent in soya and potatoes. Efficacy was 55 per cent for weeds, 31 per cent for pests and 23 per cent for diseases. On a regional basis, efficacy was 61 per cent in west Europe, 56 per cent in North America and Oceania and 37 per cent in the rest of the world. Both the potential and actual losses have increased both in actual and relative terms since the early 1960s, when yields were smaller and cropping less intensive. In 205 German wheat trials between 1985 and 1990 losses owing to diseases increased from 11 per cent when the attainable yield was 4 t ha^−1^ to 20 per cent with 11 t ha^−1^. This makes the important point that, as yields rise in the future, so too will the need for excellent crop protection. The major threats to crop protection in the future are the incidence of new resistances in the pathogens and the availability of chemical and genetic controls.

In the past, resistances have arisen where a mode of pathogen control has been used repeatedly and without recourse to alternatives. It has happened with aphids in glasshouses and is happening now with repeated use of glyphosate in rotations of herbicide-resistant crops. This will continue to occur unless there is effective regulation to prevent it. Resistance need not be a serious worry so long as there is a ready supply of alternative crop-protection products or practices that can be applied when the need arises. There is a serious risk that this may not always be so.

Chemical controls are available for most of the major weeds and airborne pests and diseases of the major crops. Farmers should be able to cope with most of the major airborne threats if these chemicals, or their replacements, remain available. However, within Europe at least, there is strong pressure to reduce the use of crop-protection chemicals and to reduce the types that can be marketed, often with little consideration of the real risk that they pose to human or animal health or the wider environment. If this trend continues, it could have serious implications for future crop yields.

The crop-protection chemistry that is available to combat soil-borne pests and diseases is less effective. If applied at all, the chemicals usually have a less-than-perfect toxicology and have to be applied to the soil in large doses. Hence, many soil-borne pathogens are currently held in check by crop rotation alone. These pathogens will become an increasing threat as the soil gets warmer and increases their multiplication rates: control by crop rotation will become less effective. Plant breeding for resistance or tolerance to these problems has been successful in a few cases in the past, but screening lines to find sources of resistance is expensive and time-consuming. It also slows progress in breeding for yield increases, as was evident while tolerance to rhizomania was introduced into sugar beet cultivars. Transgenic approaches may be the way to solve these problems. Certainly, an approach like this will be needed to prevent nematode-induced disorders and fungal root-rots from getting much worse.

Will companies that make crop-protection chemicals continue to invest in research and development as they have done in the past? The market value of these products, in real terms, fell by 18 per cent between 1998 and 2003 (Clough 2005), partly as a result of the use of genetically modified (GM) crops. It seems clear that the large agrochemical companies will continue to invest in GM, where the market is growing, and are unlikely to *expand* their activity in new crop-protection chemicals, especially as environmental concerns continue to be raised in relation to these products. Eventually, we will have to decide whether we want GM or old chemistry.

Unlike some of the problems of crop nutrition, where poverty prevents access to fertilizers, the most damaging biotic problems are weeds, and farmers in less developed countries often have access to cheap sources of labour that are every bit as effective at controlling weeds as expensive herbicides. In some of the complex and intensive cropping systems used in many developing countries, the problems caused by carry-over effects of some herbicides would make their use counter-productive. Serious difficulties will arise if labour becomes so scarce or so expensive that manual weed control is no longer possible.

### (d) The yield gap

Achievable yield of crop is defined here as the yield that could be produced by combination of the best germplasm with the best management and in an environment with the current average radiation, temperature and rainfall. It is assumed that the texture of the soil and the ability to irrigate cannot be changed (the provision of an irrigation system is not a short-term farm-management action). There is usually a large difference between the achievable yield and the commercial yield of a crop. This may be estimated as the difference between a benchmark set by a crop model (which usually simulates an experiment where agronomy is optimized) and a farm or national average. Alternatively, it can be estimated as the difference between yields from crops grown under near-perfectly managed conditions, as in variety tests, and the yields of farm or national crops grown nearby in the same season. These differences are referred to here as the yield gap. Closing this gap has huge effects on productivity and resource use efficiency. How can real-world farmers today achieve yields closer to the potential of current crop varieties, given that they cannot modify soil texture or increase the water supply?

Hochman *et al*. (2009) describe relationships between yield simulations made with a crop-growth model for 334 crops of wheat in Australia and farmers' observations of the yields of the same crops. Farmers' observations were taken from either yield monitors on the combines or from sales records. On average farmers recorded yields that were 80 per cent of the benchmark value and most of the variation in yield could be accounted for by considering evapotranspiration alone, so the effects of the farmer choosing an insufficient N fertilizer dose or an inappropriate sowing date were small. Australian farmers have access to inputs that they consider justified on economic grounds and in the wheat example there was evidence that, with hindsight, their use of N fertilizer was lavish. Also their fields are large so errors owing to the difference between field and cropped areas were small. Despite this, about 20 per cent of the benchmark value was not being harvested or sold. Only a small portion of this could be owing to losses during the harvest.

**Figure 4.**
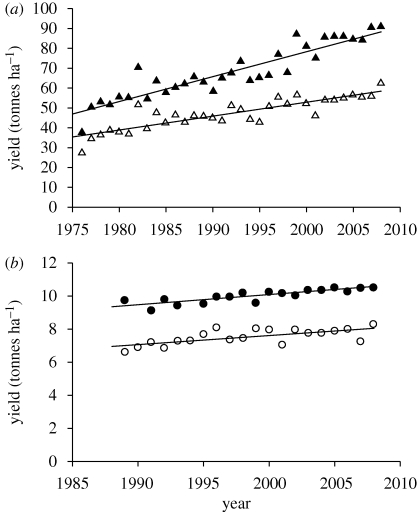
Yields of (*a*) sugar beet and (*b*) wheat in official variety tests in the UK and national average yields in the same year. Data sources are http://statistics.defra.gov.uk, www.hgca.com ((*a*); open triangle, commercial; filled triangle, variety trial and (*b*) open circle, commercial; filled circle, variety trial).

The yield gap for wheat and sugar beet crops in England and Wales is illustrated in figure 4 as the difference between official variety tests and national average yields. Wheat yields have been rising steadily and the gap has remained at about 2.3 t ha^−1^. Beet yields have been rising rapidly, but the gap has been widening; this situation is almost exactly mirrored in data from Germany, where the gap in sugar yield in the last two decades has averaged 3.5 t ha^−1^ (Märlander *et al*. 2003).

It is often assumed that these gaps are owing to lack or inappropriate use of resource by the farmers. In some places this may be true, but the gaps are also caused by:

— The inevitability that crop plants will fail to establish in a few places.
— Occasional weeds will compete for light and water.
— Spots with insufficient or excess nutrients.
— Predation by pests and loss of efficiency owing to disease.
— Inefficiencies at harvest.
— Extreme weather events that cause crop failure: floods, frosts, hail and strong winds, prolonged droughts and heat waves are all likely to become more frequent.

Scott & Jaggard (1992) tried to analyse the causes of the beet yield gap and, in addition to inefficiencies owing to seedling establishment failure and the presence of weeds and diseases, they attributed it to differences between crop and field area, early harvest while the crop was still growing rapidly, and losses during harvesting, prolonged storage and loading for delivery to the factory. Some of these gaps (i.e. early harvest and loading losses) cannot be closed if the whole industry is to remain efficient.

Yield gaps of approximately 20 per cent are common in developed countries. For example, the gaps between variety tests and the state yields for wheat and maize in Kansas between 2004 and 2007 ranged from 0.65 to 0.91 and averaged 0.71 for wheat and 0.81 for maize (data from Kansas State University). Pidgeon *et al*. (2001) estimated the sizes of yield gap for sugar beet production across Europe during the 1990s using a crop-growth model. At one extreme, France, Belgium, Netherlands and UK delivered approximately 75 per cent of the achievable yield while Poland only delivered 30 per cent. Polish sugar beet yields have risen by about 60 per cent in the last 15 years. This clearly illustrates the effects that rewards, appropriate trading arrangements, and access to modern varieties and machines can have on productivity.

Differences between achievable and actual crop yields are sometimes assessed on the basis of global agro-ecological zones (Bruinsma 2003; Kindred *et al*. 2008). For example, Bruinsma (2003) show the difference between actual and agro-ecologically attainable yields of wheat for 15 countries. The UK, France and Denmark all produce more than the amount that is apparently attainable while the USA appears to produce about half of the attainable yield. These agro-ecological zones are too crude for this type of analysis. For example, France and UK are in the same zone and should have the same attainable yields but analyses with crop-growth models show that, for beet, achievable yields are about 15 per cent more in France than in UK because the weather is more favourable.

**Figure 5.**
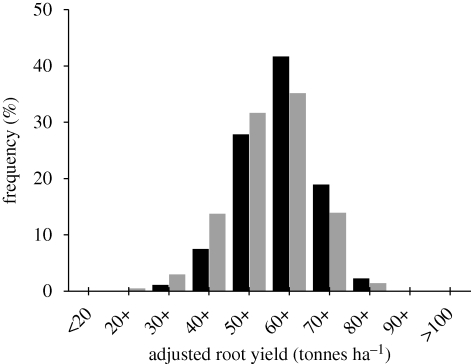
Frequency distribution of five-year average (2004–2008) adjusted root yields of sugar beet contracts, classified as percentages of all growers or as percentages of all tonnage delivered to British Sugar factories. Data from British Sugar plc (filled black bar, % tonnes; filled grey bar, % growers).

Despite the apparent stability of the yield gap, it can be narrowed. This is illustrated by the fact that neighbouring farmers can have very different yields. The distribution in the five-year average yields for all sugar beet growers in England is shown in figure 5. The highest yielders are performing almost as well as the variety tests, while the yields of the poor performers are less than half. This difference has little connection to differences in soil type or region, although it is loosely correlated with the crop's access to water. Neither is it connected to use of inputs because poor performers often spend more money on seeds, fertilizers and crop-protection chemicals than their more successful counterparts (Lang 2009). Clearly, anything that can be done to improve the performance of the below-average farm will have a large and inexpensive effect on productivity. Differences in beet yield performance between near neighbours were studied in Sweden (Blomquist *et al*. 2003) where a useful indicator of the farmer's expertise was the penetration resistance of the subsoil: large penetration resistances were indicative of operations that took place at inappropriate times and had deleterious effects on soil structure. Similar situations may be found commonly in mechanized agriculture anywhere.

## 6. Will yield increases be enough?

Smil (2005) estimated that by 2050 an enlarged world population with changed dietary requirements would need about 50 per cent more food for people and farm animals. By 2009 the FAO's World Expert Forum had raised this estimate to 70 per cent. We have attempted to analyse what these increased demands will mean for arable agriculture if they are to be realized solely by changing yield per hectare. We have done this by taking an approach analogous to that of Ewert *et al*. (2005) and Rounsevell *et al*. (2005). Using the yield statistics from FAO for 1961 to 2007 for a selection of major crops, we calculated linear trends as the yield changed with time for each country containing a zone illustrated in figure 1, except that the whole of the EU was used instead of England and Germany. The linear trends were converted to relative yield changes: the future change in yield was calculated from the relative change at the end of the observation period i.e. between 2006 and 2007. Where yield has declined (sugar cane in South Africa, Brazil and Australia, wheat in Ukraine and Nigeria) we assumed that the decline could be stopped and the relative yield change was set at zero. We assumed that each crop would react to [CO_2_] changes as described in §2: where there is no crop-specific data we used the average value for the C3 crops or the C4 crops, as appropriate. The assumed values are shown in table 5. All crops were assumed to suffer the ozone-induced yield reduction described in §3: where a crop had no specific value, we assumed the mean reduction of 7.5 per cent (this may be optimistic). Future climate change impacts in the absence of a CO_2_ effect (table 6) were either taken from crop-specific publications or were means of values (table 2) taken from Nelson *et al*. (2009). We then calculated the possible yield of various crops assuming three future yield improvement scenarios (*f*_T,P_, table 5). The first assumed that potential yield continues to improve at 1 per cent each year (i.e. current yield trends are maintained). Case 2 assumed that yield trends are modified to 70 per cent of the recent annual gain (Ewert *et al*. 2005), while scenario 3 assumed that the present trends are increased to 2 per cent per year, in line with expert opinions cited by Kindred *et al*. (2008). The current fraction of achievable yield (*f*_TG_) was assumed to be 55–80% and was assumed to increase by 10 per cent with the stimulus that is likely to be provided by extra demand for food. The proportions used in individual cases were decided according to Ewert *et al*. (2005) for wheat, Masutomi *et al*. (2009) for rice and Jaggard *et al*. (2007) for sugar beet, with the remaining crops equal to sugar beet. A summary of the most conservative projections to 2050 is shown in table 7: the others are available as the electronic supplementary material.

**Table 5.** Values for changes, by 2050, in yield (%) owing to the effects of CO_2_ and O_3_ concentrations, the present and future gain in potential yield ( *f*_T,P_) with low, current and high gain rates and current and future percentages for the yield gap (*f*_T,G_).

crop	CO_2_	O3	current (*f*_T,P_)	2050 (*f*_T,P_)			current (*f*_T,G_)	2050 (*f*_T,G_)
				low	current	high		
wheat	15.0	−9.0	1	0.7	1	2	80	90
maize	0	−5.0	1	0.7	1	2	75	85
rice	10	−4.0	1	0.7	1	2	55	65
soya bean	15.0	−12.0	1	0.7	1	2	75	85
sorghum	0	−7.5	1	0.7	1	2	75	85
dry bean	13.3	−7.5	1	0.7	1	2	75	85
rape seed	13.3	−7.5	1	0.7	1	2	75	85
sugar cane	0	−7.5	1	0.7	1	2	75	85
sugar beet	8	−7.5	1	0.7	1	2	75	85
potato	36.0	−7.5	1	0.7	1	2	75	85
sunflower	13.3	−7.5	1	0.7	1	2	75	85

**Table 6.** Assumed values of yield changes (%) as a consequence of climate change between 2007 and 2050 (no CO_2_ fertilizing effect) under the emission scenario A1B. Values are based on Nelson *et al*. (2009); Ewert *et al*. (2005); Jones *et al*. (2003) and Da Silva *et al*. (2008). The asterisk indicates where a given crop is not grown.

crops	EU	Argentina	Australia	Brazil	Canada	China	India	Nigeria	Russia	S. Africa	Ukraine	USA
wheat	−2.5	−1.3	2.8	−1.3	2.8	−1.3	−1.3	−1.3	−1.3	−1.3	−1.3	2.8
maize	−2.5	−0.3	−2.5	−0.3	−2.5	−0.3	−0.3	−0.3	−0.3	−0.3	−0.3	−2.5
rice	−4.5	−16.5	−4.5	−16.5	*	−16.5	−16.5	−16.5	−16.5	−16.5	−16.5	−4.5
soya bean	−3.2	−6	−3.5	−6	−2.5	−6	−6	−6	−6	−6	−6	−3.5
sorghum	−3.2	−6	−3.5	−6	−2.5	−6	−6	−6	−6	−6	−6	−3.5
dry bean	−3.2	−6	−3.5	−6	−2.5	−6	−6	−6	−6	−6	−6	−3.5
rape seed	−3.2	−6	−3.5	−6	−2.5	−6	−6	−6	−6	−6	−6	−3.5
sugar cane	*	9	9	9	*	9	9	9	*	9	*	9
sugar beet	0	*	*	*	0	0	*	*	0	*	0	0
potato	0	0	0	0	0	0	0	0	0	0	0	0
sunflower	−3.2	−6	−3.5	−6	−2.5	−6	−6	−6	−6	−6	−6	−3.5

**Table 7.** The factor by which yields are predicted to increase between 2007 and 2050, and the predicted 2050 yields of selected crops in selected countries/regions on the low improvement scenario. The asterisk indicates where a given crop is not grown.

crop	yield (tonnes ha^−1^)	country/region											
		EU	Argentina	Australia	Brazil	Canada	China	India	Nigeria	Russia	S. Africa	Ukraine	USA
wheat	increase factor	1.51	1.46	1.35	1.61	1.42	1.73	1.62	1.05	1.60	1.72	1.05	1.39
	2050 yield	8.4	3.7	2.4	3.2	3.5	7.9	4.8	1.8	3.1	4.3	2.8	4.0
maize	increase factor	1.42	1.57	1.49	1.51	1.27	1.55	1.41	1.32	0.95	1.40	1.91	1.36
	2050 yield	9.9	9.8	8.5	4.8	10.1	8.8	2.7	2.1	2.6	3.9	7.5	12.7
rice	increase factor	1.30	1.29	1.27	1.46	*	1.41	1.39	0.90	1.98	1.15	1.69	1.38
	2050 yield	8.4	7.3	11.0	4.6	*	9.8	4.5	1.5	8.4	2.9	7.6	10.4
sorghum	increase factor	1.37	1.43	1.22	1.08	*	1.48	1.21	1.19	1.41	1.44	1.68	1.11
	2050 yield	8.0	7.0	3.2	2.3	*	6.7	1.1	1.5	1.9	4.2	3.1	4.7
soya bean	increase factor	1.64	1.44	1.49	1.47	1.22	1.44	1.42	1.57	1.79	1.55	1.75	1.34
	2050 yield	5.0	4.0	3.3	3.8	3.2	2.7	1.6	1.2	1.9	2.7	2.3	3.7
dry bean	increase factor	1.80	1.17	1.52	1.13	1.29	1.40	1.32	*	1.93	1.35	1.39	1.25
	2050 yield	2.7	1.4	1.6	0.7	2.5	2.0	0.6	*	3.1	1.8	2.4	2.4
rape seed	increase factor	1.39	*	1.44	*	1.38	1.61	1.53	*	1.89	1.91	1.36	1.27
	2050 yield	4.3	*	2.0	*	2.2	2.8	1.6	*	2.3	2.1	1.6	2.0
sugar cane	increase factor	*	1.28	1.12	1.37	*	1.35	1.33	1.02	*	1.02	*	1.02
	2050 yield	*	87.7	98.6	104.0	*	92.6	95.6	34.3	*	72.4	*	82.7
sugar beet	increase factor	1.39	*	*	*	1.36	1.71	*	*	1.97	*	1.74	1.21
	2050 yield	79.5	*	*	*	67.0	55.5	*	*	55.4	*	42.8	62.8
potato	increase factor	1.58	1.86	1.77	1.91	1.62	1.57	1.83	1.29	1.60	1.92	1.62	1.69
	2050 yield	40.9	55.4	64.2	38.9	49.7	23.0	31.8	9.0	19.3	61.8	19.4	73.4
sunflower	increase factor	1.20	1.55	1.42	1.51	1.43	1.40	1.0	*	1.48	1.40	1.0	1.29
	2050 yield	2.0	2.9	1.5	2.3	2.4	2.6	0.6	*	1.6	1.8	1.2	2.0

In the conservative scenario (table 7) the assumptions for most crop–country combinations provide 50 per cent more yield per unit area in 2050 than in 2007. The exceptions tend to be in Russia and Ukraine, where recent changes to the political system and to rural society have caused upheaval. However, European farmers investing there expect that the productivity of arable agriculture will improve rapidly. Surprisingly, another problem is sugar cane, where yields do not seem to be improving, even in Australia where the knowledge transfer schemes within the sugar industry are second to none. The most serious cause for concern is in Africa, where we have assumed that the yield decline in Nigeria can be halted (and this is far from certain), where drought is likely to get worse if for no other reason than the growing season will be hotter, and where farming is so unprofitable that the resources needed to make improvements cannot be afforded. The scenarios with faster future growth assumptions suggest that yields per unit area will increase by about 75 per cent or will double, producing more than enough food on a global scale, although not in every region.

Although, on our assumption, yields might improve enough to feed the mean estimates of world population by 2050, there is very little room for complacency or for alternative uses for high-quality land. This review has not considered the production of bio-fuel or natural fibres like cotton, but there could be serious competition for the land resource if they are planned to occupy more land in major food-producing areas. In many areas where bio-fuel already supplies much of the energy (parts of rural China, India, large parts of Africa) there is the risk that already insufficient organic matter (OM) is returned to the soil to prevent soil degradation. This food—versus—bio-fuel question needs to be the subject for research, especially because some bio-fuel production systems are long-term investments.

## 7. Unanswered questions

This review has not addressed four important questions. The first is the extent to which degradation of the soil resource and its ability to be productive is continuing around the world. The principal causes are soil erosion (wind and water) and salinization (build-up of salts in the surface layers of the soil to reach toxic concentrations, owing to inappropriate irrigation and fertilizing practices). Both these problems have the potential to rapidly degrade what would otherwise be very productive sites. These problems tend to occur when the weather in the locality is extreme and these conditions could become more frequent in the future climate. Methods to greatly reduce the risks of erosion and salinization are well-known, but their acceptance by farmers is usually poor because the costs of putting them into practice are continuous while the need for protection is usually sporadic. The uncertainties surrounding the current extent of these problems and their future impact are large and were reviewed by Bruinsma (2003).

The second and third problems are more insidious. In many underdeveloped and developing countries agricultural products have been exported for decades, often without the soil's nutrients being replaced. For example, large quantities of material produced in Southeast Asia and exported for animal feed has led to the phosphate surplus in parts of western Europe. Whether the mining of soils for nutrients is causing reductions in productivity has not been considered here, but it is a practice that is not sustainable.

Agriculture may expand onto fresh land, sometimes because the climate changes and it becomes suitable for crop growth. This can be an important avenue for increased food production in some parts of the world (Fisher *et al*. 2005). When land is first cultivated some of the OM is oxidized to produce CO_2_. Cultivation speeds up this process, and recently reclaimed land loses OM quickly. Eventually (more than 50 years), the soils reach a stable OM state, but in this condition they are usually more difficult to manage productively. We have not attempted to assess the possible impacts of these changes.

The fourth problem is irrigation. Crop-growth models often make the assumption that irrigated crops do not suffer water shortages. This is seldom true because irrigation is usually far from perfect. The extent to which the area of irrigated cropping will be adequately supplied with water in future has not been considered. The area that receives the precipitation is seldom the area that is irrigated, and the lag time between precipitation and use of the water may be years, not months. Some of these issues as they relate to the timing of flows in major rivers like the Ganges and the Danube have been considered by Gornall *et al*. (2010).

## 8. Research and development needs

This review contains many assumptions that represent our best estimates, and some unanswered questions. Some of these assumptions should be placed on firmer footings by research and reviews aimed at the issues that are set out below.

— Detailed modelling studies of the effects of climate change (especially reduced precipitation) on yield in the presence of extra CO_2_ and its effect on water consumption. Current models can do this, but the researchers will need access to large quantities of daily weather data from many crop-production locations around the world. These studies should include potential bio-fuel and major fibre crops.
— FACE experiments with major crops to determine simultaneously the effects of elevated [CO_2_], [O_3_] and warmer temperatures on yield and water consumption.
— Studies to determine the real size of the yield gap for important crops in important crop locations. It is not sufficient that these be based on agro-ecological zone yield potential—it is too crude. Instead the potential needs to be based on benchmarks derived from crop-growth model studies or on performance in recent, official, variety tests.
— Studies to determine what can be done to close the yield difference between high-yield and low-yield farmers.
— Experiments to devise ways to supply protein-rich crops with additional N without leaving large N residues in the soil.

It is quite clear that a large part of the required yield increases must come from improved application of technologies that are likely to become available in the future (Phillips 2010). This improvement will require continued or expanded expenditure on agronomic studies to present the new technologies in formats that farmers can use profitably, and extension services to ensure that the messages get through so that yields increase without serious detriment to the environment. This will require many well-trained extension personnel.

## 9. Conclusion

By 2050 the [CO_2_] is likely to be approximately 550 ppm and FACE experiments show that this will increase yields of C3 crops by about 13 per cent but will not increase the yields of C4 species. It will also decrease water consumption, making rain-fed crops less prone to water stress. However, by then most places will be hotter by 1–3°C. This will speed up the development of existing crops, increasing the yields of indeterminate species that do not flower before harvest (such as sugar beet) and potentially decreasing the yields of determinate types like wheat and rice. The temperature rise will also increase the rate of evapotranspiration, tending to counteract the beneficial effect of CO_2_ on water consumption. This will be especially serious in those places that are already short of water. However, the changed temperature regime will also present opportunities for agronomists and plant breeders to modify cropping systems to deliver yield improvements by matching varieties to lengthened growing seasons or adopting new crop types, and this is seldom factored into yield projections. Along with changes to [CO_2_], the [O_3_] is likely to increase, especially where there is intense industrialization. This will reduce yields by at least 5 per cent.

These changes are small in comparison to the challenge ahead and in comparison to increases in crop productivity achieved in the last 50 years. To increase yield by the required amounts farmers will need improved varieties of crop plants with larger potential yields, better tolerance or resistance to pests and diseases, and more efficient extraction and use of water and nutrients. Our assumptions about future possibilities are based on past performance and they are therefore rather uncertain, but no more so than the output of some of the large climate change impact studies that rely almost entirely on multiple simulations.

There is some evidence that plant breeders are approaching a yield ceiling with the world's major crops, but the smaller-than-expected yield increases of C3 species measured in response to extra CO_2_ indicate that there are many improvements still to be made. At the same time, farmers in the developed world have good access to fertilizers and crop-protection chemicals and should be in a position to prevent serious degradation of their soil and to control weeds, pests and diseases. However, in a warmer world, soil-borne pests and diseases are likely to become more damaging: chemical control of these problems has been unsuccessful in the past. Transgenic approaches to plant breeding are likely to be needed if robust control of these problems is to be provided.

There is almost always a gap between the achievable yield of an agronomic system and the yield that is actually delivered. Part of this gap is inevitable; it relates to the way land areas are reported, to minor inefficiencies at harvest, losses during crop storage and during transport. Extreme events, like savage storms and floods, also cause part of the yield gap. These are predicted to become more frequent in the future climate. Nevertheless, even with the best agricultural extension services in developed countries, there are still large differences in performance between neighbouring farmers. It should be possible to close this gap, and there is an even larger opportunity to close the gap in those places where it is more difficult for farmers to make use of the technology that is, in theory at least, available to them.

Our assumptions and calculations indicate that it will be possible to increase food production by 50 per cent by 2050. However, this relies heavily on improved technology. Huge increases in crop yields have been made in recent decades, and the same advances cannot be repeated without major changes in crop genetics, introducing novel or foreign genes with large effects on yield. Therefore in future we will be very reliant on the maintenance of soil fertility and control mechanisms for pests, diseases and weeds, but we will be especially reliant on successful plant breeding, So long as plant breeding efforts are not hampered and modern agricultural technology continues to be available to farmers, it should be possible to produce yield increases that are large enough to meet some of the predictions of world food needs, even without having to devote more land to arable agriculture. Whether that food will be available to and affordable by all those who need it is another question.
